# Reduced Anti-Histone Antibodies and Increased Risk of Rheumatoid Arthritis Associated with a Single Nucleotide Polymorphism in PADI4 in North Americans

**DOI:** 10.3390/ijms20123093

**Published:** 2019-06-25

**Authors:** Aisha M. Mergaert, Mandar Bawadekar, Thai Q. Nguyen, Laura Massarenti, Caitlyn L. Holmes, Ryan Rebernick, Steven J. Schrodi, Miriam A. Shelef

**Affiliations:** 1 Department of Medicine, University of Wisconsin-Madison, Madison, WI 53705, USA; mergaert@wisc.edu (A.M.M.); mandar.bawadekar@gmail.com (M.B.); nguyen45@wisc.edu (T.Q.N.); cherndon@medicine.wisc.edu (C.L.H.); reberya@gmail.com (R.R.); 2 Department of Pathology and Laboratory Medicine, University of Wisconsin-Madison, Madison, WI 53705, USA; 3 Institute for Inflammation Research, Center for Rheumatology and Spine Diseases, Copenhagen University Hospital, Rigshospitalet, DK-2100 Copenhagen, Denmark; laura.massarenti@regionh.dk; 4 Center for Precision Medicine Research, Marshfield Clinic Research Institute, Marshfield, WI 54449, USA; schrodi.steven@marshfieldresearch.org; 5 William S. Middleton Memorial Veterans Hospital, Madison, WI 53705, USA

**Keywords:** rheumatoid arthritis, neutrophil extracellular trap, autoantibody, peptidylarginine deiminase 4, single nucleotide polymorphism

## Abstract

Autoantibodies against citrullinated proteins are a hallmark of rheumatoid arthritis, a destructive inflammatory arthritis. Peptidylarginine deiminase 4 (PAD4) has been hypothesized to contribute to rheumatoid arthritis by citrullinating histones to induce neutrophil extracellular traps (NETs), which display citrullinated proteins that are targeted by autoantibodies to drive inflammation and arthritis. Consistent with this theory, PAD4-deficient mice have reduced NETs, autoantibodies, and arthritis. However, PAD4′s role in human rheumatoid arthritis is less clear. Here, we determine if single nucleotide polymorphism rs2240335 in *PADI4*, whose G allele is associated with reduced PAD4 in neutrophils, correlates with NETs, anti-histone antibodies, and rheumatoid arthritis susceptibility in North Americans. Control and rheumatoid arthritis subjects, divided into anti-cyclic citrullinated peptide (CCP) antibody positive and negative groups, were genotyped at rs2240335. In homozygotes, in vitro NETosis was quantified in immunofluorescent images and circulating NET and anti-histone antibody levels by enzyme linked immunosorbent assay (ELISA). Results were compared by *t*-test and correlation of rheumatoid arthritis diagnosis with rs2240335 by Armitage trend test. NET levels did not significantly correlate with genotype. G allele homozygotes in the CCP^−^ rheumatoid arthritis group had reduced anti-native and anti-citrullinated histone antibodies. However, the G allele conferred increased risk for rheumatoid arthritis diagnosis, suggesting a complex role for PAD4 in human rheumatoid arthritis.

## 1. Introduction

Rheumatoid arthritis is an autoimmune destructive arthritis with a lifetime risk of 2.6% [1]. Most rheumatoid arthritis patients generate autoantibodies against citrullinated proteins. These anti-citrullinated protein antibodies (ACPAs) are diagnostic as measured by the anti-cyclic citrullinated peptide antibody (anti-CCP) test and pathologic [2,3]. Although there may be multiple sources of citrullinated antigens in rheumatoid arthritis, neutrophil extracellular traps (NETs), extracellular structures composed of chromatin and antimicrobial proteins, have been hypothesized to be a significant source based on observations that NETs are increased in rheumatoid arthritis and contain some of the same citrullinated proteins targeted by ACPAs [4,5,6].

Citrullination, the post-translational modification of arginines to citrullines, is catalyzed by the peptidylarginine deiminases (PADs). In activated neutrophils, PAD4 citrullinates histones enhancing chromatin unraveling and NET formation [7,8], and PAD4 is required for NETosis in response to a variety of stimuli [7,9,10,11,12]. Some of the earliest and most common ACPAs recognize citrullinated histones [13,14,15], sometimes more so if the histone is citrullinated by PAD4 as opposed to PAD2 [16]. In a murine model of rheumatoid arthritis, total IgG levels, autoantibodies, and arthritis are reduced in the absence of PAD4 [17]. These data suggest that PAD4 might contribute to the development of rheumatoid arthritis via enhanced NETosis and increased autoantibodies against histones driving disease. However, there is also data that would refute such a theory. Despite the importance of PAD4 for NETosis and the theory that NETs provide citrullinated antigen in rheumatoid arthritis, citrullination is not reduced in the absence of PAD4 in serum, lungs, or joints in murine inflammatory arthritis [9,17,18]. Moreover, the importance of PAD4 in human rheumatoid arthritis is unclear.

Understanding the role of a protein in human disease is challenging. One method for approaching this problem is to seek genetic variants associated with disease. Genome-wide association studies have identified several single nucleotide polymorphisms (SNPs) in *PADI4*, the gene encoding PAD4, that are associated with rheumatoid arthritis [19,20,21]. SNP rs2240335 is particularly interesting, since the G allele is associated with reduced PAD4 levels in neutrophils in Europeans [22]. Interestingly, the G allele of rs2240335 is associated with increased rheumatoid arthritis risk in East Asians [19,20], the opposite of what would be expected based on lower PAD4 levels in Europeans and the requirement for PAD4 in murine arthritis [17,22,23]. To date, SNP rs2240335 has not been identified as associated with rheumatoid arthritis risk in Europeans in genome-wide association studies, and no studies have evaluated rs2240335, PAD4-related biological phenomena, and rheumatoid arthritis risk together in a single human cohort.

The objective of this study is to determine if rs2240335 is associated with NETosis, anti-histone antibodies, and rheumatoid arthritis risk in a North American cohort to clarify the role of PAD4 in human rheumatoid arthritis. We found that the G allele of rs2240335 does not correlate with NETs, but does correlate with reduced anti-histone antibodies and increased risk of rheumatoid arthritis, suggesting that PAD4 has a complex role in human rheumatoid arthritis.

## 2. Results

**Figure 1:**
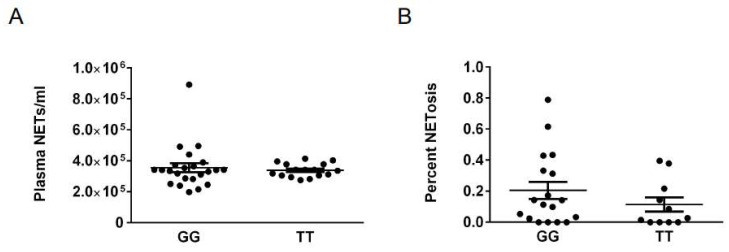
Genotype at rs2240335 does not significantly correlate with NET levels. (**A**) Circulating NET levels measured in the plasma of control subjects by ELISA were compared for homozygotes for the G and T alleles at rs2240335 by *t*-test. (**B**) Images of Sytox-stained peripheral blood neutrophils from control subjects allowed to NET for 4 h were analyzed for percent NETosis and compared for homozygotes for the G and T alleles at rs2240335 by t-test. For all panels, mean and standard error of the mean (SEM) are graphed. No comparisons are significant. For ELISA, *n*= 23 GG and *n* = 16 TT. For in vitro NETosis, *n* = 18 GG and *n* = 11 TT.

Given the association of the G allele of rs2240335 with reduced PAD4 in human neutrophils [22] and the role for PAD4 in NETosis [7], we evaluated if the rs2240335 GG genotype would correlate with reduced NET formation in a North American cohort. Since rheumatoid arthritis [4,5,6] and potentially rheumatoid arthritis medications could affect NETosis, we evaluated NETs in control subjects homozygous for the G and T alleles of rs2240335. We assessed circulating NET levels by ELISA and percent NETosis in images of Sytox-stained neutrophils allowed to undergo NETosis in vitro using a semi-automated quantification tool [6]. We detected no reduction in either circulating NETs (Figure 1A) or in vitro NETosis (Figure 1B) in subjects homozygous for the G allele at rs2240335, suggesting that rs2240335 does not correlate with NET formation in our cohort.

**Figure 2:**
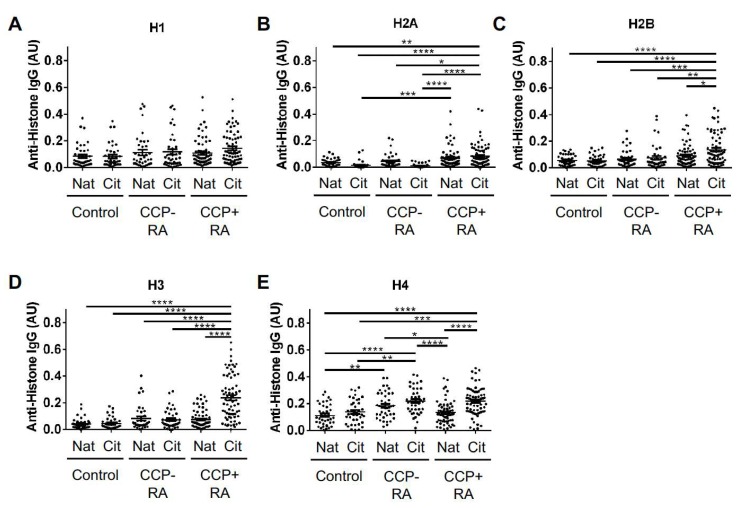
Anti-histone IgG levels in control and rheumatoid arthritis subjects. IgG levels against native (Nat) and citrullinated (Cit) histone H1 (**A**), histone H2A (**B**), histone H2B (**C**), histone H3 (**D**), and histone H4 (**E**) were measured by ELISA for controls, CCP^−^ rheumatoid arthritis (RA) and CCP^+^ RA. Graphs depict average absorbance values in arbitrary units (AU) with SEM. Groups were compared by ANOVA. For all graphs, *n* = 39 controls, 41 CCP^−^ RA, 70 CCP^+^ RA; *p < 0.05, **p < 0.01, ***p < 0.001, ****p < 0.0001.

We then sought to identify a correlation between rs2240335 and anti-histone antibody levels, since autoantibodies are reduced in arthritic mice in the absence of PAD4 [17]. Prior to this evaluation, we quantified anti-native and anti-citrullinated histone antibody levels by ELISA in control, CCP^−^ rheumatoid arthritis, and CCP^+^ rheumatoid arthritis subjects, in order to choose which group would be ideal for our experiments. We found that antibodies against histone H1 were not significantly different between control and rheumatoid arthritis subjects (Figure 2A). However, rheumatoid arthritis subjects had increased levels of antibodies against the other histones (Figure 2B–E). More specifically, autoantibody levels were increased against both native and citrullinated histone H2A in CCP^+^ subjects, with no significant increase in CCP^−^ rheumatoid arthritis (Figure 2B). As shown in Figure 2C,D, CCP^+^ subjects had higher levels of anti-citrullinated histone H2B and H3 antibodies. Anti-citrullinated histone H4 antibodies in both CCP^−^ and CCP^+^ subjects were significantly increased compared to controls (Figure 2E). Also, CCP^−^ subjects had increased antibodies to native histone H4 compared to controls.

**Figure 3:**
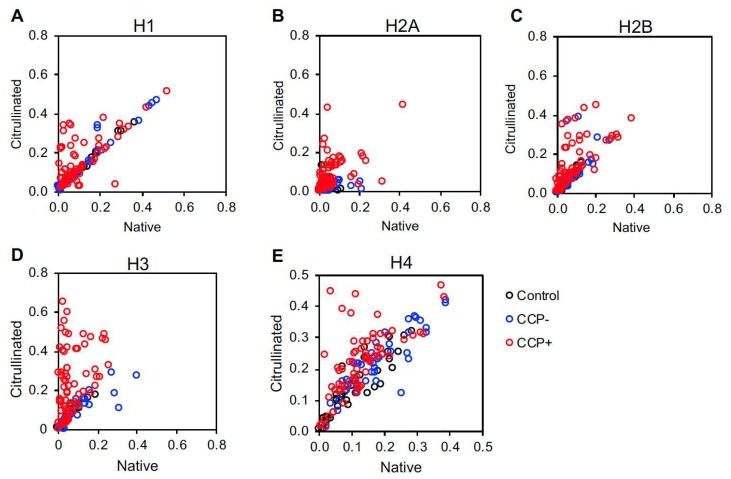
Native versus citrullinated histone autoantibody targeting in controls and rheumatoid arthritis. IgG levels against native and citrullinated histone H1 (**A**), histone H2A (**B**), histone H2B (**C**), histone H3 (**D**) and histone H4 (**E**) measured by ELISA for controls, CCP^−^ and CCP^+^ rheumatoid arthritis (RA) were compared by plotting anti-citrullinated histone antibody levels on the Y axis and anti-native histone antibody levels on the X axis. For all panels, *n* = 39 controls, *n* = 41 CCP^−^ RA, *n* = 70 CCP^+^ RA.

We also plotted each subject’s titers against native versus citrullinated histones to identify a preference for citrullinated versus native histones in individual subjects. CCP^+^ subjects had a tendency towards reactivity against the citrullinated form of all five histones (Figure 3). However, a few CCP^+^ subjects targeted native histone H2A (Figure 3B). Also, a few CCP^−^ subjects targeted native histone H2A and H3 (Figure 3B,D) and citrullinated histone H2B (Figure 3C).

**Figure 4:**
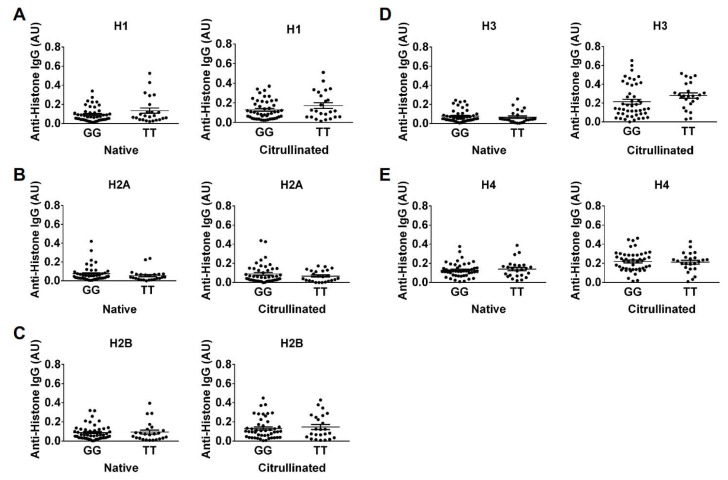
Anti-histone IgG levels in CCP^+^ rheumatoid arthritis do not significantly correlate with genotypes at rs2240335. IgG levels against native and citrullinated histone H1 (**A**), histone H2A (**B**), histone H2B (**C**), histone H3 (**D**) and histone H4 (**E**) measured by ELISA in CCP^+^ rheumatoid arthritis subjects homozygous for the G or T allele at rs2240335 were compared by t-test. For all panels, graphs depict average absorbance values in arbitrary units (AU) ± SEM, no comparisons were significant, and *n* = 46 GG and 24 TT.

**Figure 5:**
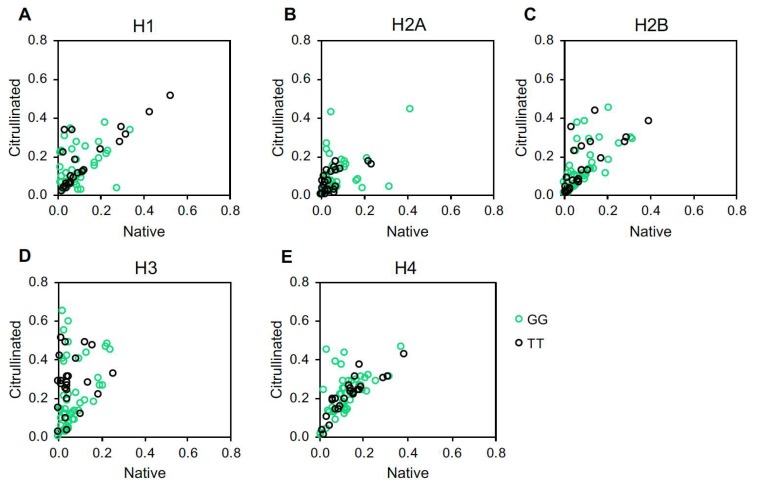
Native versus citrullinated histone autoantibody targeting in CCP^+^ rheumatoid arthritis. IgG levels against native and citrullinated histone H1 (**A**), histone H2A (**B**), histone H2B (**C**), histone H3 (**D**), and histone H4 (**E**), measured by ELISA for CCP^+^ rheumatoid arthritis subjects homozygous at rs2240335 were compared by plotting anti-citrullinated histone antibody levels on the Y axis and anti-native histone antibody levels on the *X* axis. For all panels, *n* = 46 GG and 24 TT.

Given the strong preference for citrullinated histones in CCP^+^ subjects, as expected, we compared serum IgG levels against citrullinated and native histones for CCP^+^ subjects homozygous for the G versus T allele of rs2240335. We found that there is no significant difference in anti-histone IgG between the GG versus TT genotypes in CCP^+^ subjects (Figure 4). Moreover, when plotting autoantibody levels against native versus citrullinated histones for each group, GG and TT subjects generally clustered similarly with a few GG subjects seeming to be outliers with increased autoantibodies to citrullinated and native histone H2A and citrullinated histone H4 (Figure 5).

**Figure 6:**
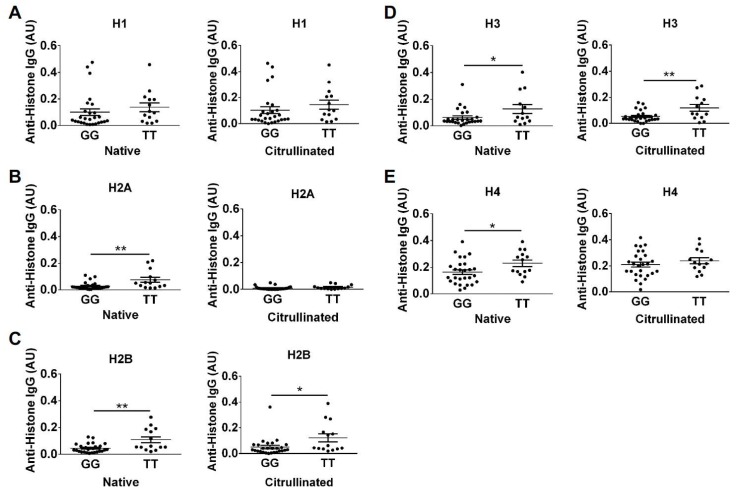
Reduced anti-histone IgG levels in CCP^−^ rheumatoid arthritis subjects homozygous for the G allele at rs2240335. IgG levels against native and citrullinated histone H1 (**A**), histone H2A (**B**), histone H2B (**C**), histone H3 (**D**), and histone H4 (**E**), measured by ELISA in CCP^−^ rheumatoid arthritis subjects homozygous for the G or T allele at rs2240335 were compared by t-test. For all panels, graphs depict average absorbance values in arbitrary units (AU) ± SEM; *p < 0.05, **p < 0.01; *n* = 27 GG and *n* = 14 TT.

**Figure 7:**
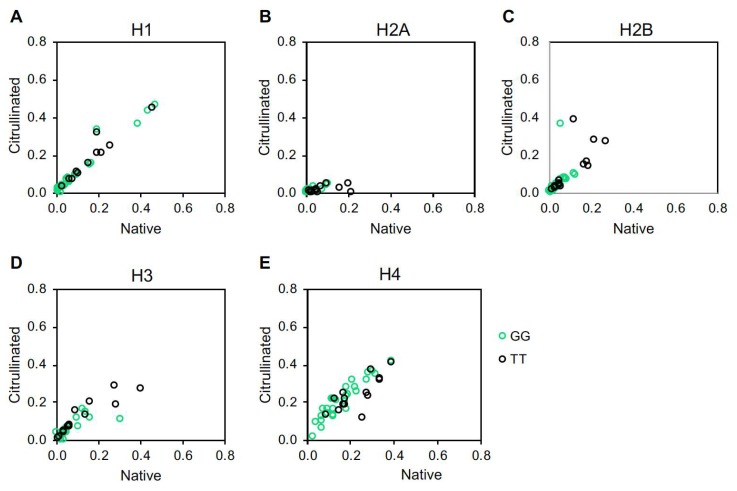
Autoantibodies against native versus citrullinated histones in CCP^−^ subjects. IgG levels against native and citrullinated histone H1 (**A**), histone H2A (**B**), histone H2B (**C**), histone H3 (**D**), and histone H4 (**E**), measured by ELISA for CCP^−^ rheumatoid arthritis subjects homozygous at rs2240335 were compared by plotting anti-citrullinated histone antibody levels on the Y axis and anti-native histone antibody levels on the *X* axis. For all panels, *n* = 27 GG and *n* = 14 TT.

Surprised by the results for the CCP^+^ subjects, we theorized that high levels of ACPAs, which are known to be cross-reactive [15,24,25,26], may have obscured effects of rs2240335. Since some CCP^−^ subjects were reactive to histones in our study, we hypothesized that these subjects might have detectable differences in anti-histone antibody levels between the homozygous alleles of rs2240335. Therefore, we compared anti-histone antibody levels measured by ELISA between subjects homozygous for the G versus T alleles of rs2240335. We found that CCP^−^ subjects homozygous for the G allele had reduced antibodies against native histones H2A, H2B, H3, and H4 and citrullinated histones H2B and H3 (Figure 6). Total IgG levels in homozygotes for the G allele of rs2240335 were not reduced (Supplementary Figure S2). When plotting native versus citrullinated titers for each genetic group, the TT and GG subjects clustered similarly with the majority of subjects showing no preference for citrullinated or native histone and a few TT subjects showing preference for native histone H2A (Figure 7). Together, these results suggest that the G allele of rs2240335, which is associated with reduced levels of PAD4 in neutrophils [22], is also associated with reduced levels of anti-histone antibodies in CCP^−^ rheumatoid arthritis.

Finally, as noted above, the G allele of rs2240335 was associated with rheumatoid arthritis risk in East Asian populations [19,20], yet reduced PAD4 levels were associated with the G allele in Europeans [22]. Therefore, we determined if rs2240335 is associated with rheumatoid arthritis in our North American cohort. For the 110 control and 452 White rheumatoid arthritis subjects in the University of Wisconsin Rheumatology Biorepository (94% of total subjects), we found that rs2240335 was associated with the rheumatoid arthritis/control endpoint using the Armitage trend test (*p* = 0.03), where the G allele conferred increased risk for rheumatoid arthritis diagnosis compared to the T allele. Comparing the GG and TT homozygous genotypes, the odds ratio was calculated to be 2.14 with 95% CI (1.11, 4.12).

## 3. Discussion

Given the association between the G allele of rs2240335 in *PADI4* and increased rheumatoid arthritis risk in East Asians [19,20], the reduction of PAD4 levels associated with the G allele of rs2240335 in Europeans [22], the role of PAD4 in NETosis, autoantibodies, and arthritis in mice [7,17], and the link between NETs, ACPAs, and human rheumatoid arthritis [4,27], we determined if rs2240335 would be associated with NETs, anti-citrullinated histone antibodies, and rheumatoid arthritis in a North American cohort. Our data suggest that the relationships between rs2240335, biologic processes involving PAD4, and rheumatoid arthritis are quite complex.

For example, we detected no significant difference in NET levels between GG and TT homozygotes at rs2240335. There are several possible explanations for this finding. First, unlike the loss of NETs in the absence of PAD4 in PAD4^−/−^ mice [7], the reduction in PAD4 levels associated with rs2240335 may not be sufficient to reduce NETosis. Also, recent reports suggest that PAD4 is not required for the formation of NETs in response to some stimuli, particularly stimuli that do not induce citrullination [28,29,30,31]. Thus, perhaps the NETosis that we measured simply does not require PAD4. Finally, our North American cohort may not be identical to the European cohort, in which PAD4 was reduced in association with rs2240335 [22]. However, this last possibility is unlikely since 94% of our North American cohort is white and overwhelmingly reported Northwestern European ancestry similar to the European cohort [22]. Additionally, when analyses were repeated for NET and anti-histone antibody experiments using only white subjects, results were almost identical.

In contrast to the lack of correlation between rs2240335 and NETs, we found that CCP^−^ subjects homozygous for the G allele at rs2240335 had reduced autoantibodies against histones (Figure 6). There was no preference for binding to citrullinated versus native histones in the majority of subjects regardless of genotype (Figure 7), consistent with negative anti-CCP testing. The mechanism underlying these findings is unknown. Given the observed normal NET levels in the GG homozygotes (Figure 1), it is unlikely that reduced anti-histone antibody levels are due to a loss of NETs. Also, the reduction in anti-native histone antibodies suggests that the mechanism does not relate to an effect of rs2240335 in *PADI4* on citrullinated antigen. Consistent with this conclusion, no loss of gross protein citrullination was detected in arthritic PAD4-deficient mice [9,17,18]. Interestingly, the reduced anti-histone antibodies seen in the GG homozygotes at rs2240335 is similar to the reduced autoantibody levels against native and citrullinated antigens including histone H2B in PAD4-deficient mice [17], although GG homozygotes did not show a similar loss of total IgG (Supplementary Figure S2). Together these findings suggest a role for PAD4 in autoantibody development apart from providing citrullinated antigen.

We also found that the G allele at rs2240335 was associated with increased rheumatoid arthritis risk, although somewhat weakly, in North American whites. Although this finding in North Americans agrees with findings in East Asians, increased rheumatoid arthritis risk associated with the G allele would not be predicted to co-associate with reduced PAD4 in neutrophils [22] or reduced anti-histone antibodies (Figure 6). Thus, there may be other effects of the G allele of rs2240335 on the development of rheumatoid arthritis. For example, the G allele of rs2240335 is associated with increased PAD4 expression in monocytes [22]. Although monocytes and macrophages are known to express PAD4 [21,22,32,33], the function of PAD4 in this lineage is essentially unknown. Monocytes and macrophages can also make extracellular traps [34,35] and thus could be a source of citrullinated antigen that is potentially PAD4-dependent. Additionally, rs2240335 could have effects in other cell types given the role of PAD4 in regulating gene expression [36,37], modulating p53 effects [38], and regulating hematopoietic progenitor cells [39]. Further studies are needed to determine how rs2240335 might drive rheumatoid arthritis in PAD4-dependent or PAD4-independent pathways. Such studies will be important to understand disease pathophysiology and to inform the development of PAD4 inhibitors as potential treatments for rheumatoid arthritis.

The association between the GG homozygotes at rs2240335 with reduced anti-histone antibodies and increased rheumatoid arthritis risk also highlights a potential disconnect between autoantibodies and disease. While it is possible that the reduction of autoantibodies in GG homozygotes was too small to affect rheumatoid arthritis pathogenesis, another possibility is that not all autoantibodies are pathologic. Studies have demonstrated a pathologic role for autoantibodies against a few citrullinated antigens [2,3]. The autoantibodies that were reduced in GG homozygotes did not specifically target citrulline, raising the possibility that pathogenicity lies primarily in ACPAs. Alternatively, autoantibodies may not be pathogenic in CCP^−^ rheumatoid arthritis.

Finally, a peripheral contribution of our study is a detailed analysis of autoantibodies against all histones in CCP^−^ and CCP^+^ rheumatoid arthritis. Multiple studies have identified citrullinated histones as autoantibody targets in rheumatoid arthritis [13,14,15]. Moreover, peroxynitrite-modified, 16α-hydroxyestrone-adducted, and acetylated histones have also been identified as autoantigens [40,41,42]. Although antibodies have been detected against native histones in rheumatoid arthritis [42,43], our study provides a comprehensive evaluation of autoantibodies against all 5 histones, including both citrullinated and native variants in both CCP^−^ and CCP^+^ rheumatoid arthritis. The reactivity seen against native histone H2A (Figure 2 and Figure 3) highlights the targeting of native antigens in rheumatoid arthritis, a commonly overlooked phenomenon. Further, the reactivity against histone H4 in CCP^−^ subjects (Figure 2) who also tested negative for rheumatoid factor (Supplementary Table S1) supports the theory that not all seronegative disease is truly seronegative. Together, these findings are useful for understanding the diversity of reactivity against histones and also reveal novel features of the autoantibody repertoire in rheumatoid arthritis.

## 4. Materials and Methods

### 4.1. Human Subjects

All subjects gave their informed consent for inclusion before they participated in the study. The study was conducted in accordance with the Declaration of Helsinki, and the protocol was approved by the Institutional Review Board of the University of Wisconsin-Madison (#2015-0156). All clinical data and biologic samples were obtained from the University of Wisconsin (UW) Rheumatology Biorepository first described in [6,44]. Briefly, the biorepository contains data and samples from subjects at least 18 years old and medically homed at UW Health. Rheumatoid arthritis subjects were identified by having at least two outpatient visits with rheumatoid arthritis associated International Classification of Diseases (ICD) codes within 24 months [45] or one visit and a positive anti-CCP test. Diagnosis was confirmed by manual review of rheumatology notes in the electronic medical record. Two categories of rheumatoid arthritis subjects were selected: those with a negative anti-CCP test result (CCP^−^) and those with an anti-CCP test result that was twice the upper limit of normal (CCP^+^). Anti-CCP titers were determined using the Immunoscan CCPlus test kit (Eurodiagnostika, Malmö, Sweden) according to the manufacturer’s instructions or generation II anti-CCP or anti-CCP3 ELISA (Inova, San Diego, CA, USA) in the UW Health clinical lab. Controls were excluded if they had an autoimmune disease, inflammatory disease, or hematologic malignancy. Control subjects, CCP^−^ rheumatoid arthritis subjects, and CCP^+^ rheumatoid arthritis subjects had similar demographic features for the GG versus TT genotype, except for smoking in the CCP^+^ rheumatoid arthritis group (Supplementary Table S1).

### 4.2. DNA Preparation

Peripheral blood was collected into ethylenediaminetetraacetic acid (EDTA) vacutainers (Becton, Dickinson and Company, Franklin Lakes, NJ, USA). DNA was isolated using the Gentra Puregene Blood Kit (Qiagen, Venlo, Netherlands) according to the manufacturer’s instructions and stored at −80 °C until use.

### 4.3. Plasma Preparation

Peripheral blood was collected into EDTA vacutainers, centrifuged at 2000× *g* for 10 min at room temperature without braking. Plasma was transferred to a new tube, centrifuged at 2000× *g* for 5 min at room temperature with braking, transferred to a new tube, and stored at −80 °C until use.

### 4.4. Genotyping

At the University of Wisconsin-Madison Biotechnology Center, DNA concentration was verified with Quant-iT PicoGreen dsDNA kit (Life Technologies, Grand Island, NY, USA). For the KASPar polymerase chain reaction (PCR) reaction, DNA samples were standardized to 0.5 ng/µL using epMotion 5075 and 10 mM Ultrapure Tris-HCl pH 7.5 (Life Technologies). A volume of 2 µL of 0.5 ng/µL DNA and 2 µL of KASPar reaction mix (480 µL KASP V4.0 2X Mastermix with standard ROX (KBioSciences, Hoddesdon, United Kingdom) and 13.2 µL assay mix (12 µM FWD Primer 5′-GAAGGTGACCAAGTTCATGCTCCCATGCAGGTACCATCACG-3′, 12 µM REV Primer 5′-GAAGGTCGGAGTCAACGGATTACCCCATGCAGGTACCATCACT-3′, 30 µM Common Primer 5′-AAGGAACAGAGGCCTGAAGGAGTTT-3′, and 4.6 mM Tris HCl pH 7.5)) were dispensed into a 384 dark-well plate (Bio-Rad Microseal PCR plates, Bio-Rad, Hercules, CA, USA). After brief centrifugation, 10 µL of Bio-Rad Chill-Out Liquid Wax was added and the following amplification protocol was used with an Eppendorf Mastercycler pro384. First, samples were incubated at 94 °C for 15 min. Then, a three-step protocol was repeated 20 times: 94 °C for 10 s, 59 °C for 5 s, 72 °C for 10 s. Then, another three-step protocol was repeated 25 times: 94 °C for 10 s, 59 °C for 20 s, 72 °C for 40 s, and finally at 10 °C until analysis. To determine the products of the PCR reaction, the plate was stored in darkness at room temperature until the wax melted and became transparent. Then a Synergy 2 (BioTek, Winooski, USA) plate reader measured fluorescence using Gen5 software (Biotek).

### 4.5. NET Enzyme Linked Immunosorbent Assay (ELISA)

Similar to a previous NET ELISA assay [46], Costar 96-well high binding ELISA plates (Corning, NY, USA) were coated overnight at 4 °C with 5 μg/mL anti-human myeloperoxidase (clone 4A4, Bio-Rad) diluted in 1X coating buffer from the Cell Death Detection ELISA kit (MilliporeSigma, Burlington, MA, USA). Then, plates were washed four times with wash buffer (0.2% Tween-20 in PBS), blocked with 5% non-fat dry milk in PBS for 2 h at room temperature, washed four times, and incubated overnight at 4 °C with plasma samples diluted 1:50 in 1% BSA in PBS. Wells were then washed four times, incubated for 2 h at room temperature with anti-DNA-POD (Cell Death Detection ELISA kit, MilliporeSigma) diluted 1X in 1% BSA in PBS, washed four times, incubated with 1-Step Ultra TMB-ELISA Substrate Solution (Thermo Fisher Scientific, Waltham, MA, USA) for 10 min, then stopped with 0.2 N H_2_SO_4_. Absorbance was read at 450 nm with 540 nm plate correction, using a Synergy 2 plate reader (BioTek) and normalized to a neutrophil NET standard. The neutrophil NET standard was generated by incubating a known number of purified neutrophils with phorbol 12-myristate 13-acetate and ionomycin overnight at 37 °C with 5% CO_2_. After 100% NETosis was visualized, NETs were scraped from the plate and stored at −80 °C.

### 4.6. Quantification of In Vitro NETosis

Neutrophils were purified and then incubated for 4 h without any stimulant, followed by fixation, Sytox staining, imaging, and semi-automated quantification of NETosis using DNA Area and NETosis Analysis (DANA) as previously [6].

### 4.7. Citrullination of Recombinant Human Histone

Recombinant human histones H1, H2A, H2B, H3 (New England Biolabs, Ipswich, UK) and H4 (MilliporeSigma) were citrullinated with recombinant human PAD4 (Cayman Chemical, Ann Arbor, MI, USA) at a ratio of 2 µg of PAD4 per mg of histone in a buffer of 100 mM Tris-HCl pH7.5, 1 mM DTT, and 5 mM CaCl_2_ at room temperature overnight with citrullination confirmed (Supplementary Figure S1).

### 4.8. Anti-Histone ELISA

Corning 96 well plates were left uncoated or were coated with 10µg/mL of native or citrullinated histone, or the same concentration of PAD4 present in the citrullinated histone solution in phosphate buffered saline (PBS) overnight at 4 °C. After washing 3 times (0.1% Tween 20 in PBS), wells were incubated with block solution (5% nonfat dried milk in 0.2% Tween 20 in PBS) at room temperature for 2–4 h, then with serum (prepared as in [44]) diluted 1:200 in block solution for 2 h at room temperature. Wells were then washed 5 times, incubated with anti-human IgG-HRP diluted 1:5000 in 5% nonfat dried milk in 0.2% Tween 20 in PBS for 2 h at room temperature, washed again 5 times, and exposed to 1-Step Slow TMB-ELISA (Thermo Fisher Scientific) for 5–10 min, then stopped with 0.18 M sulfuric acid. Plates were read at 450 nm with 540 nm plate correction using a Synergy 2 plate reader (BioTek). Samples were run in duplicate. Absorbance values from uncoated wells for each sample were subtracted from coated wells for each sample to reduce the effects of non-specific IgG binding to the plastic. Absorbance values from PAD4 coated wells were subtracted from citrullinated histone coated wells to normalize for anti-PAD4 IgG binding.

### 4.9. Statistical Analysis

T-tests and analysis of variance (ANOVA) were performed using Prism (GraphPad Software, San Diego, CA, USA) with *p* < 0.05 considered statistically significant. The Armitage trend test with permutation routine was calculated using the XLISP-STAT programming language; 1 M iterations of the permutation were performed to obtain a permuted *p*-value.

## 5. Conclusions

We demonstrate that rs2240335 in *PADI4* correlates with reduced anti-histone antibodies and increased rheumatoid arthritis risk in North Americans. Taken together with the work of others, our data suggest that PAD4 may play a complex role in human rheumatoid arthritis. Additionally, we have provided a detailed evaluation of anti-histone antibodies in CCP^−^ and CCP^+^ rheumatoid arthritis.

## Supplementary Materials

Supplementary materials can be found at https://www.mdpi.com/1422-0067/20/12/3093/s1. **Table S1:** Characteristics of Rheumatoid Arthritis (RA) and Control Subjects. **Figure S1:** Citrullination of Histones. **Figure S2:** Total Serum IgG levels in CCP^−^ Rheumatoid Arthritis Subjects.

## Abbreviations

ACPA	anti-citrullinated protein antibody
CCP	cyclic citrullinated peptide
NET	neutrophil extracellular trap
PAD4	peptidylarginine deiminase
SNP	single nucleotide polymorphism
