# Supplementary material for: Reduced Anti-Histone Antibodies and Increased Risk of Rheumatoid Arthritis Associated with a Single Nucleotide Polymorphism in PADI4 in North Americans
Source: Int J Mol Sci. 2019 Jun 25;20(12):3093. doi: 10.3390/ijms20123093 (PMC6627847; doi:10.3390/ijms20123093)
Supplement: Supplementary file 1 [file ijms-20-03093-s001.pdf]

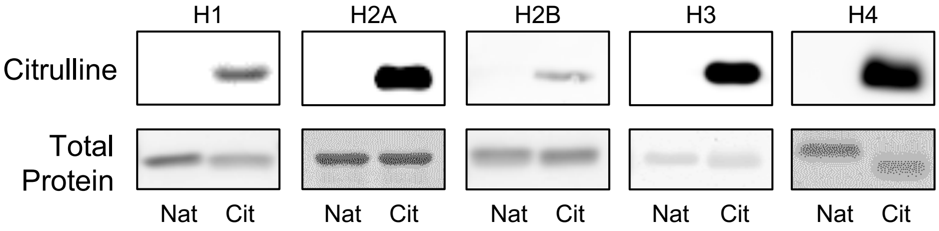

Supplementary Figure 1. Citrullination of Histones. Recombinant human histones H1, H2A, H2B, H3, and H4 were incubated in buffer alone (Nat) or citrullinated with PAD4 in buffer (Cit). 2μg of each histone was run by SDS-PAGE for total protein visualization by Coomassie staining and blotting for visualization of citrullination using the anti-citrulline (modified) detection kit (MilliporeSigma) according to manufacturer's instructions. Blots are representative of at least 5 replicates.

Table S1. Characteristics of Rheumatoid Arthritis (RA) and Control Subjects

	Control			CCP-			CCP+		
	GG (n=23)	TT (n=16)	p	GG (n=27)	TT (n=14)	p	GG (n=46)	TT (n=24)	p
Age, mean years (SE)	49.5 (3.8)	53.1 (4.6)	0.55	59.3 (2.4)	56.3 (3.6)	0.47	61.2 (1.8)	58.2 (2.3)	0.31
Sex, female (%)	19 (82.6)	14 (87.5)	0.68	20 (74.1)	11 (78.6)	0.75	34 (73.9)	18 (75.0)	0.92
Body Mass Index, mean (SE)	31.5 (1.7)	26.1 (2.4)	0.07	28.7 (1.3)	28.6 (2.4)	0.97	30.0 (0.8)	30.6 (1.6)	0.70
Smoking Status, number (%)									
Current smoker	1 (4.4)	2 (12.5)	0.20	3 (11.1)	0 (0.0)	0.41	**3 (6.5)**	**5 (20.8)**	**0.03**
Former smoker	3 (13.0)	5 (31.3)		9 (33.3)	6 (42.9)		21 (45.7)	4 (16.7)	
Never smoked	19 (82.6)	9 (56.3)		15 (55.6)	8 (57.1)		22 (47.8)	15 (62.6)	
Charlson Comorbidity Score, mean (SE)	1.6 (0.4)	1.4 (0.4)	0.67	4.0 (0.6)	3.7 (0.6)	0.79	3.6 (0.3)	3.3 (0.4)	0.48
Race/Ethnicity, number (%)									
White	21 (91.3)	13 (81.3)	0.61	22 (81.5)	12 (85.7)	0.85	40 (87.0)	17 (70.8)	0.30
Black	0 (0.0)	0 (0.0)		1 (3.7)	1 (7.1)		1 (2.2)	3 (12.5)	
Hispanic	0 (0.0)	0 (0.0)		1 (3.7)	0 (0.0)		2 (4.4)	2 (8.3)	
Native American	1 (4.4)	1 (6.3)		1 (3.7)	1 (7.1)		2 (4.4)	2 (8.3)	
Asian	1 (4.4)	2 (12.5)		1 (3.7)	0 (0.0)		0 (0.0)	0 (0.0)	
Pacific Islander	0 (0.0)	0 (0.0)		0 (0.0)	0 (0.0)		0 (0.0)	0 (0.0)	
Other	0 (0.0)	0 (0.0)		1 (3.7)	0 (0.0)		1 (2.2)	0 (0.0)	
Rheumatoid Factor Positive, number (%)	-	-	-	5 (71.4)	2 (28.6)	0.59	33 (67.4)	16 (32.7)	0.70
Erosive Disease, number (%)	-	-	-	12 (54.6)	10 (45.5)	0.10	22 (75.9)	7 (24.1)	0.13
Time Since RA Diagnosis, mean years (SE)	-	-	-	20.0 (3.3)	13.1 (2.7)	0.18	14.9 (1.8)	11.8 (2.0)	0.28

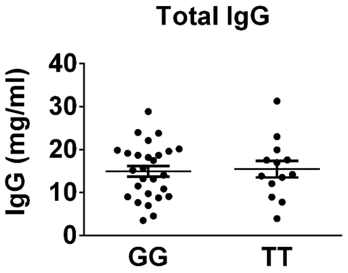

Supplementary Figure 2. Total Serum IgG levels in CCP- Rheumatoid Arthritis Subjects. Total IgG was quantified in CCP- rheumatoid arthritis subjects using the Human IgG Total ELISA Ready-SET-Go! Kit (Thermo Fisher Scientific) according to the manufacturer’s instructions. Serum IgG levels were compared between homozygotes for the G and T alleles of rs2240335 by t test with the mean ± SEM graphed. No significant difference was seen. n=27 GG and n=13 TT.
